# Epigenetic Transgenerational Modifications Induced by Xenobiotic Exposure in Zebrafish

**DOI:** 10.3389/fcell.2022.832982

**Published:** 2022-02-25

**Authors:** Luis Terrazas-Salgado, Alejandra García-Gasca, Miguel Betancourt-Lozano, Raúl Llera-Herrera, Isabel Alvarado-Cruz, Beatriz Yáñez-Rivera

**Affiliations:** ^1^ Centro de Investigación en Alimentación y Desarrollo, A. C., Mazatlán, Mexico; ^2^ Instituto de Ciencias del Mar y Limnología—Unidad Académica Mazatlán, Universidad Nacional Autónoma de México, Mazatlán, Mexico; ^3^ Department of Cellular and Molecular Medicine, University of Arizona Cancer Center, Tucson, AZ, United States; ^4^ Consejo Nacional de Ciencia y Tecnología, México, Mexico

**Keywords:** epigenetics, Zebrafish, xenobiotic, transgenerational, exposure

## Abstract

Zebrafish (*Danio rerio*) is a well-established vertebrate model in ecotoxicology research that responds to a wide range of xenobiotics such as pesticides, drugs, and endocrine-disrupting compounds. The epigenome can interact with the environment and transform internal and/or external signals into phenotypic responses through changes in gene transcription. Environmental exposures can also generate epigenetic variations in offspring even by indirect exposure. In this review, we address the advantages of using zebrafish as an experimental animal model to study transgenerational epigenetic processes upon exposure to xenobiotics. We focused mostly on DNA methylation, although studies on post-translational modifications of histones, and non-coding RNAs related to xenobiotic exposure in zebrafish are also discussed. A revision of the methods used to study epigenetic changes in zebrafish revealed the relevance and reproducibility for epigenetics-related research. PubMed and Google Scholar databases were consulted for original research articles published from 2013 to date, by using six keywords: zebrafish, epigenetics, exposure, parental, transgenerational, and F2. From 499 articles identified, 92 were considered, of which 14 were selected as included F2 and epigenetic mechanisms. Current knowledge regarding the effect of xenobiotics on DNA methylation, histone modifications, and changes in non-coding RNAs expressed in F2 is summarized, along with key experimental design considerations to characterize transgenerational effects.

## Introduction

Zebrafish (*Danio rerio;* Cyprinidae) is a small freshwater teleost, which presents several advantages as a model organism such as short generation time, high fecundity, transparent and *ex utero* embryonic development, and high genetic homology to humans (Santos et al., 2017). These characteristics support the widespread use of zebrafish for environmental transgenerational epigenetic studies. The zebrafish genome exhibits high levels of global DNA methylation, with 7–8% methylated cytosines from a 36% GC content in adults (Han and Zhao, 2008). Likewise, active developmental enhancers are hypermethylated in zebrafish DNA; something that has not been observed in other species (Kamstra et al., 2017). In addition, eight mammalian orthologs DNA methyl-transferase (DNMT) enzymes are expressed in zebrafish (Kamstra et al., 2015). Recent advances in genome editing techniques based on zinc finger nuclease, transcription activator-like effector nuclease (TALEN), and the highly successful clustered regularly interspaced short palindromic repeats (CRISPR-Cas) technique have changed the speed at which single gene functions can be addressed in this model during the last decade (López Nadal et al., 2020). Currently, several studies have characterized DNA methylation profiles (Skvortsova et al., 2019), histone modifications, and non-coding RNAs (Best et al., 2018), providing relevant information on the dynamics of epigenetic regulation in zebrafish.

**FIGURE 1:**
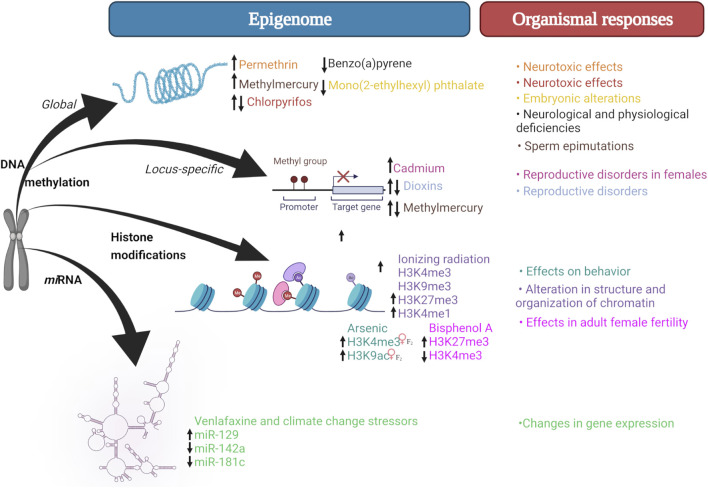
Epigenetic mechanisms triggered by exposure to xenobiotics. Xenobiotic exposure may affect different epigenetic mechanisms, which in turn may result in phenotypic (e.g., neurotoxic, reproductive, behavioral) alterations. Created with BioRender.com.

While effects of xenobiotic exposure have been reported in zebrafish (Figure 1), only a few studies examine their transgenerational consequences in F2 or subsequent generations (Table 1). In this regard, it is important to differentiate between intergenerational and transgenerational inheritance, which conceptually differs between teleost fish and mammalian models. In pregnant female mammals, subsequent generations are directly exposed to xenobiotics such as embryos (F1) and embryonic germ cells from F1 embryos (F2) within the parental generation (F0) (intergenerational exposure). Consequently, transgenerational effects in female mammals will be observed until F3, after which they may persist or “disappear” through subsequent generations. In teleost fish, only the F1 generation is considered intergenerational, while the F2 and F3 generations are considered transgenerational, thus, in zebrafish, F2 is equivalent to the exposure-free F3 in the mouse (Baker et al., 2014; Best et al., 2018).

**TABLE 1:** Xenobiotic exposure studies that include F2 and epigenetic approaches in zebrafish. A: adult, E: embryo, L: larvae exposure, WL: whole larvae.

Xenobiotic	Exposure	Transgenerational Evaluation	Tissue	Differentially expressed gene	Epigenetic process					Organismal responses (F2)	Reference
					DNA methylation		Histone modifications		*nc*RNA		
	Stage/Time (d)				Global	Site-specific	Methylation	Acethylation			
Heavy metals									
Arsenic	E **150**	F2	WL		n-s					Heart rate increased. Larval behavior alterations. Increase anxiety behavior	Valles et al. (2020)
			brain	*bdnf,* decreased	n-s	n-s	H3K4me3, enrichment (females only)	H3K9ac, n-s
Cadmiun	E 169	F2 & F3	ovaries	*cyp19a1a,* increased (F2 only) *foxl2a*, n-s		*foxl2a*, hypomethylation (F2 only)				Progressive feminization of the population	Pierron et al. (2021)
Mercury	E 1	F2	sperm			Differential DNA methylation regions				Hyperactivity and visual deficit	Carvan et al. (2017)
Methylmercury	A 47	F2	WL	Five detoxification related genes, n-s	n-s	rRNA, hypermethylation				Olsvik et al. (2014)
Compounds derivated from hydrocarbons and other industrial processes											
Benzo [a]pyrene	E 5	F2	WL	*dnmt*, decreased (120 hpf, only F0 evaluation)	Decreased (120 hpf, only F0 evaluation)					Gender-specific increased body mass index. Increased oxygen consumption. Hyper-avoidance behavior	Knecht et al. (2017)
BPA	A 14	F2	testicular cells and sperm	*insra* and *insrb,* decreased (only F0 and F1 evaluation)	n-s (only F0 evaluation)					Malformed larvae	Lombó et al. (2015)
	A 28	F2 & F3	ovaries	*amh*, decreased		hypermethylation	H3K4me3, enrichment H3K27me3, enrichment			Body mass and gonadosomatic indexes, and fertility rate	Santangeli et al. (2019)
Mono(2-ethylhexyl) phthalate	E 6	F2	WL	*dnmt*, decreased (120 hpf, only F0 evaluation)	n-s	24 CpG sites				Non significant reduction of larval body length	Kamstra et al. (2017)
2,3,7,8-tetrachlorodibenzo-p-dioxin	L <1	F2	testicular	19 genes related to epigenetic regulation	n-s	Differential DNA methylation regions and sites				Defects in spermatogenesis and decreased percentage of fertilized eggs	Akemann et al. (2020)
Venlafaxine	A 21	F2	liver	hsp90 and hsp70, increased					miR-181c-5p and miR-16c-5p, n-s		Luu et al. (2021)
Pesticides											
Chlorpyrifos-oxon	E 5	F2	WL	Three neuro-developmental related genes, n-s	Increased					Hyperactive behavior Increased acetylcholine concentration and AChE activity	Schmitt et al. (2020)
Permethrin	E 28	F2	brain	626 to 792						Locomotor and anxiety behavior	Blanc et al. (2021)
Radiation											
Gamma	E <1	F2	ovaries	*hnf4a*, increased (only F0 evaluation). *gmnn* and *vegfab*, decreased (only F0 evaluation)			H3K4me3, enrichment H3K9me3, enrichment H3K27me3, enrichment (only F1)				Lindeman et al. (2019)
Ionizing	A 27	F2 & F3	WL	41 gene clusters (only F1 evaluation)		Differential DNA methylation of five regions					Kamstra et al. (2018)

The focus of environmental epigenetics is to understand how gene regulatory mechanisms operate in the absence of genome sequence changes. The adaptive responses to xenobiotics can be inherited through different mechanisms, such as DNA methylation and histone modifications that regulate gene expression at the DNA and chromatin levels respectively, and non-coding RNAs (ncRNA), like microRNAs, that constitute a post-transcriptional mechanism that regulates the half-life and translation of specific mRNAs (Best et al., 2018; Chatterjee et al., 2018). In this context, the relevance of studies that include multiple generations (F2, F3, F4, ....., Fn) is crucial to elucidate the specific role of parental exposure, either by physiological adaptation or through disruption between generations. When xenobiotic exposure occurs, effects can be maintained, and genes can be continuously silenced or activated by different mechanisms for multiple generations (Ho and Burggren, 2010); something that can be studied to link epigenetic modifications with organismal responses, and their relation to environmental exposure. In this minireview, we summarize recent findings regarding epigenetic regulation in zebrafish by exposure to xenobiotics, addressing the main mechanisms studied in the field of zebrafish transgenerational epigenetics.

## Epigenetic Mechanisms

### DNA Methylation in Zebrafish

DNA methylation is the best-studied epigenetic mechanism. This process is defined by the covalent addition of a methyl group at carbon five of the cytosine residue (mC), and it is involved in gene regulation and genome maintenance (Goll and Halpern, 2011; Kamstra et al., 2015). Zebrafish possess multiple DNA methyl-transferase (*dnmt*) gene homologs of the mammalian housekeeping *dnmt1*, *dnmt3a* (*dnmt3a1* and *2*), and *dnmt3b* genes (*dnmt3b1*, *2*, *3*, and *4*), which are responsible for DNA methylation and demethylation processes (Kamstra et al., 2015; Cavalieri and Spinelli, 2017). When a transgenerational effect occurs, the epigenetic mark is mitotically and meiotically stable without reprogramming itself in primordial germ cells and post-fertilization embryos. The levels of 5-methylcytosine (5-mC) can be measured in a simplified “one-step” ELISA type reaction; however this footprint may be biased since not all xenobiotics produce the same effect on methylation levels, and each cell type may express a different signature throughout development and different life stages (Santangeli et al., 2019; Pierron et al., 2021). For instance, no differences were observed in F0 and subsequent generations after bisphenol-A (BPA) and arsenic (As) exposure (Santangeli et al., 2019; Valles et al., 2020), while different levels of DNA methylation were observed in different generations after exposure to chlorpyrifos (F1 and F2 showed hypomethylation, whereas F3 presented hypermethylation) probably due to a paternal and/or maternal influence (Schmitt et al., 2020). Thus, global DNA methylation may not be the most sensitive marker to assess xenobiotic-induced transgenerational effects in zebrafish.

A more informative approach could be the locus-specific evaluation of methylated bases. This strategy implies previous evaluation of gene expression to identify differentially expressed genes in response to xenobiotic exposure. For example, it has been reported that BPA exposure generates locus-specific DNA methylation changes associated with reproductive disturbances in males and females (*dnmt1*, anti-müllerian hormone (*amh*), follicle-stimulating hormone receptor (*fshr*), Sry-box transcription factor 2 (*sox2*)*,* and insulin receptor b (*insrb*) (Lombó et al., 2015; Santangeli et al., 2019). Also, cadmium (Cd) exposure showed changes in the promoter of the forkhead boxl2a (*foxl2a*) gene (which is involved in ovary maintenance; Yang et al., 2017) in female gonads from F0 to F3, resulting in the progressive feminization of the population over generations (Pierron et al., 2021). In permethrin exposure, changes of DMR in both sexes on chromatin remodeling 24 (chr24) and vimentin (vim) genes were observed, with modifications in the same sites from F0 to F2; furthermore, sex-specific differentially methylated regions (DMR) were found related to the paternal inheritance of F0 to F2 in fragile-X mental retardation protein translational regulator 1 (fmr1) and prepronociceptin b (pnocb) genes, both related to the development of the zebrafish nervous system (Blanc et al., 2021). As with permethrin, Akemann et al. (2020) observed that exposure to 2,3,7,8- tetrachlorodibenzo-p-dioxin (TCDD) in male zebrafish did not generate changes in global methylation in gonads at any generation; however, they did observe DMRs from F0 to F2. In the F1 generation, 159 genes with differential methylation and expression were identified, while in F2 there were five, pre-B-cell leukemia homeobox 3b (pbx3b; transcriptional activator), si:dkey- 266f7.5 (unknown function), snail family zinc finger 3 (snai3; roles in mesodermal formation during embryogenesis; involved in spermatogenesis), transmembrane protein 132E (tmem132e; required for normal inner ear hair cell function and hearing), and calpain 7 (capn7; member of the calpain family of proteins), all except tmem132e showed differential methylation in the three generations.

Most experimental research employs DNA methylation approaches: therefore, changes in this epigenetic mechanism are well documented throughout zebrafish embryonic development. However, it is still necessary to increase the understanding regarding the interaction of epigenetic mechanisms and how they could be altered by exposure to xenobiotics (Best et al., 2018). In F1, the inheritance of the methylation pattern has been related to gender; and recent evidence indicates that paternal exposure has an important influence on the phenotype of the offspring (Ord et al., 2020). The offspring of males exposed to xenobiotics, such as BPA, show phenotypic alterations in the expression of genes involved in cardiogenesis and the epigenetic profile (Lombó and Herráez, 2021). However, transgenerational inheritance is influenced by both maternal (Santangeli et al., 2019) and paternal (Lombó et al., 2015) methylation dynamics.

### Histone Modifications in Zebrafish

Histone structure facilitates DNA packaging and modulates access of transcription factors to different regions of the genome (Eirin-Lopez and Putnam, 2018). Chromatin structure can directly affect RNA polymerase II binding sites and other epigenetic modifiers such as DNMTs, and ultimately modify gene expression (Aluru, 2017; Cavalieri and Spinelli, 2017).

The most relevant histone post-translational modifications occur in specific amino acid residues and include acetylation, methylation, and phosphorylation; all of which are reversible (Best et al., 2018). Histone acetylation dynamics promote an open chromatin conformation favoring gene expression and this greatly affects the ε-amino group of lysine (K) residues at the N-terminus of H3 and H4*;* this is mediated by histone acetylases (HAT) and histone deacetylases (HDAC). At the same time, histone methylation can activate (i.e., trimethylation of histone H3 lysine4; H3K4me3) or repress (i.e., trimethylation of histone *H3* lysine 27; H3K27me3) gene expression. In zebrafish, evidence suggests that environmental exposure to xenobiotics induces histone hyperacetylation and hypermethylation under As exposure, showing an increased H3K4me3 in the nervous system of F0 and F2, with a reduction in neurotrophic factor expression in the brain (Valles et al., 2020). Likewise, Lindeman et al. (2019) reported that embryonic zebrafish exposure to gamma radiation (a recognized genotoxic agent) can cause epigenetic changes, generating an enrichment of H3K4me3 in the hepatocyte nuclear factor 4, alpha (*hnf4a*), geminin DNA replication inhibitor (*gmnn*), and vascular endothelial growth factor Ab (*vegfab*) loci. The authors found that F1 embryos from exposed parents showed hypermethylation of H3K4me3, H3K9me3, and H3K27me3 at the same three loci, while these differences were almost negligible in F2 embryos, suggesting that ionizing radiation can affect the structure and chromatin organization and that these changes can be detected intergenerationally (F1), but not transgenerationally (F2). Ionizing radiation can also produce genetic damage, which may in turn generate epigenetic modifications, as has been observed with 8-oxo-7,8-dihydroguanine (8-oxoG) (Hao et al., 2018; Giorgio et al., 2020). Histone demethylation due to exposure to xenobiotics has also been observed. For example, BPA in adult zebrafish during spermatogenesis promoted demethylation of H3K27me3 (Santangeli et al., 2019), although it remains unclear how histone modification profiles and transgenerational effects are related to xenobiotics. Given the crosstalk between different epigenetic mechanisms, the development of comprehensive studies on gene expression and epigenetic modifications could help to provide useful information on how xenobiotic exposure could influence gene expression through epigenetic regulation and the eventual phenotypic effects.

### ncRNA in Zebrafish

ncRNAs are transcribed and edited RNA molecules that do not code for proteins but are essential in the regulation of mRNA preventing translation or directing chromatin remodeling. These ncRNAs include microRNAs (miRNAs), small interfering RNAs (siRNAs), Piwi-associated RNAs (piRNAs), and long non-coding RNAs (lncRNAs). Although these ncRNAs are an important component of the epigenetic machinery, their role is the least documented regarding transgenerational modifications by xenobiotic exposure. Few studies have characterized the role of individual miRNAs in xenobiotic-induced phenotypic changes and their possible effects in F2. A recent study by Luu et al. (2021) reported that exposure to elevated temperatures, hypoxia, and venlafaxine in adult zebrafish caused significant decreases in miR-142a in the exposed F0 and F1 generations, as well as a significant reduction in miR-181c in F1. Interestingly, the authors report a significant inverse relationship between cytochrome P450 family 3, subfamily A, polypeptide 65 (*cyp3a65*; as a detoxification mechanism) expression and miR-142a, besides a significant reduction in miR-181c, and a decrease in the coactivator one alpha (*ppargca*), an indicator of energy stress and mitochondrial biogenesis (LeMoine et al., 2010). These changes persisted in two subsequent generations, suggesting that parental exposure to xenobiotics, as well as multiple stressors, can confer transcriptional, post-transcriptional, and epigenetic responses in F2 generations associated with ncRNAs.

## Discussion (Future Basic Research Directions and Possible Benefits of Integrative Approaches)

Exposure to a number of xenobiotics (i.e., arsenic, lead, and cadmium) may cause alterations in DNA methylation and histone modifications. In contrast, little information is available on xenobiotic-induced modifications in ncRNA (Dusinska et al., 2017).

Knowledge regarding the interaction of factors in the epigenetic machinery is key to implementing a standardized approach to evaluate transgenerational epigenetic alterations caused by xenobiotics. An important shortcoming is that most experimental studies use high concentrations and acute exposure. Thus, experimental designs resembling realistic scenarios with chronic exposures at environmental concentrations are needed to increase our comprehension of transgenerational effects under feasible conditions.

To understand epigenetic alterations due to xenobiotic exposure, differential effects between males and females need to be characterized, although the evidence is limited to maternal inheritance, specifically in genes involved in sexual determination at early stages of development (Santangeli et al., 2019). Long-term evaluation studies (at least up to F3) are necessary to assess the persistence of epigenetic marks; in this regard, the information available is scarce (only 3 publications reach F3), and the results are inconsistent, which could be related to the epigenetic mark under study and the analytical method. For instance, there are studies in which epigenetic marks (such as DNA methylation) persist until F3 (Kamstra et al., 2018), while in other studies the same epigenetic marks decrease (Pierron et al., 2021) or are not observed even in F2 (Santangeli et al., 2019). This indicates that studies must analyze in detail the possible epigenetic marks, as well as the methodological limitations. Sensitive techniques such as those based on bisulfite sequencing, ATAC-seq, and ChiP-seq may nowadays be the best way to evaluate locus-specific epigenetic marks or histone modifications (Kamstra et al., 2018; Santangeli et al., 2019).

Bisulfite-modified single-stranded DNA (with high conversion efficiency) provides a sensitive approach to identify and map 5 mC with a single base-pair resolution, (Kurdyukov and Bullock, 2016; Li, 2021). The methods for DNA methylation analysis have come a long way in recent years, where technologies based on third-generation sequencing allow the study of base modifications that include 5 mC (which is the dominant form in eukaryotes and has been recognized as the best-characterized epigenetic marker), 6 mA (more recently defined as another important epigenetic marker in higher eukaryotes) and 4mC (restricted to prokaryotes and archaea), without going through specific chemical treatments (Koziol et al., 2016; Koh et al., 2018; Gouil and Keniry, 2019; Gaultney et al., 2020; Wang et al., 2020). Similarly, DNA methylation can be determined by high throughput NGS analysis such as whole-genome bisulfite sequencing (WGBS), although the large amount of data obtained from WGBS requires robust bioinformatic analysis (Li, 2021).

The most commonly used method to determine and quantify chromatin modifications is the chromatin immunoprecipitation assay (ChIP) (Li, 2021). This technique identifies DNA-protein interactions and can be combined with other techniques to study histone modifications and the interactions with different chromatin regulators. The combination of epigenetic approaches such as DNA methylation and histone modifications provides valuable information regarding gene transcription and chromatin conformation. Also, ChIP bisulfite methylation sequencing (ChIP-BMS) allows determination of the methylation status of ChIP-DNA removed by a specific antibody (histone markers or transcription factors), providing the possibility to evaluate the interactions between histone modifications and DNA methylation, transcription factor binding, and methylation of transcription factor-binding sites (TFBS), as well as multiple interactions between genetic and epigenetic factors (Li and Tollefsbol, 2011).

ncRNAs participate as epigenetic regulators of gene expression through direct and indirect actions on chromatin (Holoch and Moazed, 2015). Some methods to study small ncRNAs include reverse transcription coupled with quantitative and digital PCR, hybridization-based methods, and high-throughput RNA sequencing (Pritchard et al., 2012). In contrast to small ncRNAs that normally mediate RNA silencing processes, lncRNAs exhibit a wide diversity of mechanisms through interaction with RNA-binding proteins (RBPs) in specific regions of DNA (Long et al., 2017); since most of them contain normal 5′-caps and 3′ poly-A tails (Sun et al., 2018), they can be detected by standard qRT-PCR; nevertheless, current high-throughput technologies, such as Chromatin isolation by RNA purification, Capture Hybridization Analysis of RNA Targets, RNA antisense purification, RNA Immunoprecipitation, Cross-linking and immunoprecipitation and RNA pull-down, may be better platforms to understand the global lncRNA profile (Cao et al., 2019).

Emphasis on the bioinformatic approaches is needed to interpret the data, extract information, and identify candidate genes affected by DNA methylation, chromatin modifications, and ncRNAs (Arora and Tollefsbol, 2021; Li, 2021). The general process for a correct bioinformatic analysis of DNA methylation data throughout the genome by NGS involves the implementation of specific protocols related to library preparation, sequencing, quality control, reading alignment, and data analysis using software for different sequencing platforms (Arora and Tollefsbol, 2021; Li, 2021). Many useful tools have been developed to analyze various types of DNA methylation sequencing data, such as the web-based genome browser *UCSC Genome Browser* (https://genome.ucsc.edu) or *Ensembl* (https://www.ensembl.org), which can be used for data visualization. Briefly, bioinformatic analysis of NGS is performed with raw datasets (generated either using single-end or pair-end sequencing), and subsequently, quality control is carried out using different software (such as FastQC, RnBeads, or Meffil). Later, the data are aligned to the reference genome using software such as BWA, Hisat2, or Bowtie. The process of calling variants is diverse and based on the experiment; different software can be used for this purpose such as SAMtools or GATK. Finally, depending on different requirements, annotation is performed using ANNOVAR, SAVANT, or SVA software (Arora and Tollefsbol, 2021; Li, 2021).

So far, experimental research has focused primarily on DNA methylation due to the functional link between epigenetic reprogramming and this epigenetic mechanism. Here is where the zebrafish model could be most useful, enabling suitable experimental approaches to investigate transgenerational epigenetic effects through integrative studies (i.e., histone modifications, non-coding RNA, and chromatin structure, along with responses at higher levels of biological organization). Altogether, this could provide a better understanding of transgenerational effects after xenobiotic exposure under realistic environmental scenarios.

## References

[B1] Akemann C. Meyer D. N. Gurdziel K. Baker T. R. (2020). TCDD-induced Multi- and Transgenerational Changes in the Methylome of Male Zebrafish Gonads. Environ. Epigenetics 6, dvaa010. 10.1093/eep/dvaa010 PMC7660120 33214906

[B2] Aluru N. (2017). Epigenetic Effects of Environmental Chemicals: Insights from Zebrafish. Curr. Opin. Toxicol. 6, 26–33. 10.1016/j.cotox.2017.07.004 29202112 PMC5703436

[B3] Arora I. Tollefsbol T. O. (2021). Computational Methods and Next-Generation Sequencing Approaches to Analyze Epigenetics Data: Profiling of Methods and Applications. Methods 187, 92–103. 10.1016/j.ymeth.2020.09.008 32941995 PMC7914156

[B4] Baker T. R. King-Heiden T. C. Peterson R. E. Heideman W. (2014). Dioxin Induction of Transgenerational Inheritance of Disease in Zebrafish. Mol. Cell Endocrinol. 398, 36–41. 10.1016/j.mce.2014.08.011 25194296 PMC4262573

[B5] Best C. Ikert H. Kostyniuk D. J. Craig P. M. Navarro-Martin L. Marandel L. (2018). Epigenetics in Teleost Fish: From Molecular Mechanisms to Physiological Phenotypes. Comp. Biochem. Physiol. B: Biochem. Mol. Biol. 224, 210–244. 10.1016/j.cbpb.2018.01.006 29369794

[B6] Blanc M. Antczak P. Cousin X. Grunau C. Scherbak N. Rüegg J. (2021). The Insecticide Permethrin Induces Transgenerational Behavioral Changes Linked to Transcriptomic and Epigenetic Alterations in Zebrafish (*Danio rerio*). Sci. Total Environ. 779, 146404. 10.1016/j.scitotenv.2021.146404 33752003

[B7] Cao M. Zhao J. Hu G. (2019). Genome-wide Methods for Investigating Long Noncoding RNAs. Biomed. Pharmacother. 111, 395–401. 10.1016/j.biopha.2018.12.078 30594777 PMC6401243

[B8] Carvan M. J. Kalluvila T. A. Klingler R. H. Larson J. K. Pickens M. Mora-Zamorano F. X. (2017). Mercury-induced Epigenetic Transgenerational Inheritance of Abnormal Neurobehavior Is Correlated with Sperm Epimutations in Zebrafish. PLOS ONE 12, e0176155. 10.1371/journal.pone.0176155 28464002 PMC5413066

[B9] Cavalieri V. Spinelli G. (2017). Environmental Epigenetics in Zebrafish. Epigenetics & Chromatin 10, 46. 10.1186/s13072-017-0154-0 28982377 PMC5629768

[B10] Chatterjee N. Gim J. Choi J. (2018). Epigenetic Profiling to Environmental Stressors in Model and Non-model Organisms: Ecotoxicology Perspective. Environ. Health Toxicol. 33, e2018015. 10.5620/eht.e2018015 30286591 PMC6182246

[B11] Dusinska M. Tulinska J. El Yamani N. Kuricova M. Liskova A. Rollerova E. (2017). Immunotoxicity, Genotoxicity and Epigenetic Toxicity of Nanomaterials: New Strategies for Toxicity Testing? Food Chem. Toxicol. 109, 797–811. 10.1016/j.fct.2017.08.030 28847762

[B12] Eirin-Lopez J. M. Putnam H. M. (2018). Marine Environmental Epigenetics. Annu. Rev. Mar. Sci. 11, 335–368. 10.1146/annurev-marine-010318-095114 29958066

[B13] Gaultney R. A. Vincent A. T. Lorioux C. Coppée J.-Y. Sismeiro O. Varet H. (2020). 4-Methylcytosine DNA Modification Is Critical for Global Epigenetic Regulation and Virulence in the Human Pathogen *Leptospira Interrogans* . Nucleic Acids Res. 48, 12102–12115. 10.1093/nar/gkaa966 33301041 PMC7708080

[B14] Giorgio M. Dellino G. I. Gambino V. Roda N. Pelicci P. G. (2020). On the Epigenetic Role of Guanosine Oxidation. Redox Biol. 29, 101398. 10.1016/j.redox.2019.101398 31926624 PMC6926346

[B15] Goll M. G. Halpern M. E. (2011). DNA Methylation in Zebrafish. Prog. Mol. Biol. Translational Sci. 101, 193–218. 10.1016/B978-0-12-387685-0.00005-6 PMC5455991 21507352

[B16] Gouil Q. Keniry A. (2019). Latest Techniques to Study DNA Methylation. Essays Biochem. 63, 639–648. 10.1042/EBC20190027 31755932 PMC6923321

[B17] Han L. Zhao Z. (2008). Comparative Analysis of CpG Islands in Four Fish Genomes. Comp. Funct. Genomics 2008, 1–6. 10.1155/2008/565631 PMC2375969 18483567

[B18] Hao W. Qi T. Pan L. Wang R. Zhu B. Aguilera-Aguirre L. (2018). Effects of the Stimuli-dependent Enrichment of 8-oxoguanine DNA Glycosylase1 on Chromatinized DNA. Redox Biol. 18, 43–53. 10.1016/j.redox.2018.06.002 29940424 PMC6019822

[B19] Ho D. H. Burggren W. W. (2010). Epigenetics and Transgenerational Transfer: a Physiological Perspective. J. Exp. Biol. 213, 3–16. 10.1242/jeb.019752 20008356

[B20] Holoch D. Moazed D. (2015). RNA-mediated Epigenetic Regulation of Gene Expression. Nat. Rev. Genet. 16, 71–84. 10.1038/nrg3863 25554358 PMC4376354

[B21] Kamstra J. H. Aleström P. Kooter J. M. Legler J. (2015). Zebrafish as a Model to Study the Role of DNA Methylation in Environmental Toxicology. Environ. Sci. Pollut. Res. 22, 16262–16276. 10.1007/s11356-014-3466-7 25172464

[B22] Kamstra J. H. Hurem S. Martin L. M. Lindeman L. C. Legler J. Oughton D. (2018). Ionizing Radiation Induces Transgenerational Effects of DNA Methylation in Zebrafish. Sci. Rep. 8, 15373. 10.1038/s41598-018-33817-w 30337673 PMC6193964

[B23] Kamstra J. H. Sales L. B. Aleström P. Legler J. (2017). Differential DNA Methylation at Conserved Non-genic Elements and Evidence for Transgenerational Inheritance Following Developmental Exposure to Mono(2-Ethylhexyl) Phthalate and 5-azacytidine in Zebrafish. Epigenetics & Chromatin 10, 20. 10.1186/s13072-017-0126-4 28413451 PMC5389146

[B24] Knecht A. L. Truong L. Marvel S. W. Reif D. M. Garcia A. Lu C. (2017). Transgenerational Inheritance of Neurobehavioral and Physiological Deficits from Developmental Exposure to Benzo[a]pyrene in Zebrafish. Toxicol. Appl. Pharmacol. 329, 148–157. 10.1016/j.taap.2017.05.033 28583304 PMC5539966

[B25] Koh C. W. Q. Goh Y. T. Toh J. D. W. Neo S. P. Ng S. B. Gunaratne J. (2018). Single-nucleotide-resolution Sequencing of humanN6-Methyldeoxyadenosine Reveals Strand-Asymmetric Clusters Associated with SSBP1 on the Mitochondrial Genome. Nucleic Acids Res. 46, 11659–11670. 10.1093/nar/gky1104 30412255 PMC6294517

[B26] Koziol M. J. Bradshaw C. R. Allen G. E. Costa A. S. H. Frezza C. Gurdon J. B. (2016). Identification of Methylated Deoxyadenosines in Vertebrates Reveals Diversity in DNA Modifications. Nat. Struct. Mol. Biol. 23, 24–30. 10.1038/nsmb.3145 26689968 PMC4941928

[B27] Kurdyukov S. Bullock M. (2016). DNA Methylation Analysis: Choosing the Right Method. Biology 5, 3. 10.3390/biology5010003 PMC4810160 26751487

[B28] LeMoine C. M. R. Lougheed S. C. Moyes C. D. (2010). Modular Evolution of PGC-1α in Vertebrates. J. Mol. Evol. 70, 492–505. 10.1007/s00239-010-9347-x 20443112

[B29] Li Y. (2021). Modern Epigenetics Methods in Biological Research. Methods 187, 104–113. 10.1016/j.ymeth.2020.06.022 32645449 PMC7785612

[B30] Li Y. Tollefsbol T. O. (2011). DNA Methylation Detection: Bisulfite Genomic Sequencing Analysis. Methods Mol. Biol. 791, 11–21. 10.1007/978-1-61779-316-5_2 21913068 PMC3233226

[B31] Lindeman L. C. Kamstra J. H. Ballangby J. Hurem S. Martín L. M. Brede D. A. (2019). Gamma Radiation Induces Locus Specific Changes to Histone Modification Enrichment in Zebrafish and Atlantic salmon. PLOS ONE 14, e0212123. 10.1371/journal.pone.0212123 30759148 PMC6373941

[B32] Lombó M. Fernández-Díez C. González-Rojo S. Navarro C. Robles V. Herráez M. P. (2015). Transgenerational Inheritance of Heart Disorders Caused by Paternal Bisphenol A Exposure. Environ. Pollut. 206, 667–678. 10.1016/j.envpol.2015.08.016 26322593

[B33] Lombó M. Herráez M. P. (2021). Paternal Inheritance of Bisphenol A Cardiotoxic Effects: The Implications of Sperm Epigenome. Ijms 22, 2125. 10.3390/ijms22042125 33672782 PMC7924642

[B34] Long Y. Wang X. Youmans D. T. Cech T. R. (2017). How Do lncRNAs Regulate Transcription? Sci. Adv. 3, eaao2110. 10.1126/sciadv.aao2110 28959731 PMC5617379

[B35] López Nadal A. Ikeda-Ohtsubo W. Sipkema D. Peggs D. McGurk C. Forlenza M. (2020). Feed, Microbiota, and Gut Immunity: Using the Zebrafish Model to Understand Fish Health. Front. Immunol. 11, 114. 10.3389/fimmu.2020.00114 32117265 PMC7014991

[B36] Luu I. Ikert H. Craig P. M. (2021). Chronic Exposure to Anthropogenic and Climate Related Stressors Alters Transcriptional Responses in the Liver of Zebrafish (*Danio rerio*) across Multiple Generations. Comp. Biochem. Physiol. C: Toxicol. Pharmacol. 240, 108918. 10.1016/j.cbpc.2020.108918 33141083

[B37] Olsvik P. A. Williams T. D. Tung H.-s. Mirbahai L. Sanden M. Skjaerven K. H. (2014). Impacts of TCDD and MeHg on DNA Methylation in Zebrafish (*Danio rerio*) across Two Generations. Comp. Biochem. Physiol. Part C: Toxicol. Pharmacol. 165, 17–27. 10.1016/j.cbpc.2014.05.004 24878852

[B38] Ord J. Heath P. R. Fazeli A. Watt P. J. (2020). Paternal Effects in a Wild‐type Zebrafish Implicate a Role of Sperm‐derived Small RNAs. Mol. Ecol. 29, 2722–2735. 10.1111/mec.15505 32525590

[B39] Pierron F. Lorioux S. Héroin D. Daffe G. Etcheverria B. Cachot J. (2021). Transgenerational Epigenetic Sex Determination: Environment Experienced by Female Fish Affects Offspring Sex Ratio. Environ. Pollut. 277, 116864. 10.1016/j.envpol.2021.116864 33714788

[B40] Santangeli S. Consales C. Pacchierotti F. Habibi H. R. Carnevali O. (2019). Transgenerational Effects of BPA on Female Reproduction. Sci. Total Environ. 685, 1294–1305. 10.1016/j.scitotenv.2019.06.029 31272786

[B41] Santos D. Luzio A. Coimbra A. M. (2017). Zebrafish Sex Differentiation and Gonad Development: A Review on the Impact of Environmental Factors. Aquat. Toxicol. 191, 141–163. 10.1016/j.aquatox.2017.08.005 28841494

[B42] Schmitt C. Peterson E. Willis A. Kumar N. McManus M. Subbiah S. (2020). Transgenerational Effects of Developmental Exposure to Chlorpyrifos-Oxon in Zebrafish (DANIO RERIO). Toxicol. Appl. Pharmacol. 408, 115275. 10.1016/j.taap.2020.115275 33049267

[B43] Skvortsova K. Tarbashevich K. Stehling M. Lister R. Irimia M. Raz E. (2019). Retention of Paternal DNA Methylome in the Developing Zebrafish Germline. Nat. Commun. 10, 3054. 10.1038/s41467-019-10895-6 31296860 PMC6624265

[B44] Sun Q. Hao Q. Prasanth K. V. (2018). Nuclear Long Noncoding RNAs: Key Regulators of Gene Expression. Trends Genet. 34, 142–157. 10.1016/j.tig.2017.11.005 29249332 PMC6002860

[B45] Valles S. Hernández-Sánchez J. Dipp V. R. Huerta-González D. Olivares-Bañuelos T. N. González-Fraga J. (2020). Exposure to Low Doses of Inorganic Arsenic Induces Transgenerational Changes on Behavioral and Epigenetic Markers in Zebrafish (*Danio rerio*). Toxicol. Appl. Pharmacol. 396, 115002. 10.1016/j.taap.2020.115002 32277946

[B46] Wang H.-T. Xiao F.-H. Li G.-H. Kong Q.-P. (2020). Identification of DNA N6-Methyladenine Sites by Integration of Sequence Features. Epigenetics & Chromatin 13, 8. 10.1186/s13072-020-00330-2 32093759 PMC7038560

[B47] Yang Y.-J. Wang Y. Li Z. Zhou L. Gui J.-F. (2017). Sequential, Divergent, and Cooperative Requirements of *Foxl2a* and *Foxl2b* in Ovary Development and Maintenance of Zebrafish. Genetics 205, 1551–1572. 10.1534/genetics.116.199133 28193729 PMC5378113

